# Liver Autophagy in Anorexia Nervosa and Acute Liver Injury

**DOI:** 10.1155/2014/701064

**Published:** 2014-08-27

**Authors:** Marouane Kheloufi, Chantal M. Boulanger, François Durand, Pierre-Emmanuel Rautou

**Affiliations:** ^1^INSERM, U970, Paris Cardiovascular Research Center (PARCC), 75015 Paris, France; ^2^Université Paris Descartes, Sorbonne Paris Cité, UMR-S970, 75015 Paris, France; ^3^DHU Unity, Service d'Hépatologie, Hôpital Beaujon, Assistance Publique-Hôpitaux de Paris, 92 110 Clichy, France

## Abstract

Autophagy, a lysosomal catabolic pathway for long-lived proteins and damaged organelles, is crucial for cell homeostasis, and survival under stressful conditions. During starvation, autophagy is induced in numerous organisms ranging from yeast to mammals, and promotes survival by supplying nutrients and energy. In the early neonatal period, when transplacental nutrients supply is interrupted, starvation-induced autophagy is crucial for neonates' survival. In adult animals, autophagy provides amino acids and participates in glucose metabolism following starvation. In patients with anorexia nervosa, autophagy appears initially protective, allowing cells to copes with nutrient deprivation. However, when starvation is critically prolonged and when body mass index reaches 13 kg/m^2^ or lower, acute liver insufficiency occurs with features of autophagic cell death, which can be observed by electron microscopy analysis of liver biopsy samples. In acetaminophen overdose, a classic cause of severe liver injury, autophagy is induced as a protective mechanism. Pharmacological enhancement of autophagy protects against acetaminophen-induced necrosis. Autophagy is also activated as a rescue mechanism in response to Efavirenz-induced mitochondrial dysfunction. However, Efavirenz overdose blocks autophagy leading to liver cell death. In conclusion, in acute liver injury, autophagy appears as a protective mechanism that can be however blocked or overwhelmed.

## 1. Introduction

Autophagy (literally “self-eating”) is a cellular process responsible for the degradation of excess or aberrant long-lived cytosolic proteins and organelles within lysosomes in order to remove and eventually recycle the resulting macromolecules [1]. It has an important role in various biological events such as cellular remodeling during development and differentiation, adaptation to stress conditions, and extension of lifespan [2]. Depending on physiological functions and mode of cargo delivery to the lysosome, three forms of autophagy have been identified: chaperone-mediated autophagy, microautophagy, and macroautophagy [3]. In this review we will focus on macroautophagy, hereafter referred to as “autophagy.”

Autophagy consists of several sequential steps by which a portion of the cytoplasm, including organelles, is engulfed by a phagophore to form an autophagosome. The autophagosome subsequently fuses with a lysosome to form an autolysosome, and the internal material is degraded by lysosomal hydrolases and recycled to the cytoplasm [4].

The initial studies that led to the identification of autophagy were conducted in the liver [5]. Afterward, extensive work has been carried out on this organ to dissect the regulation and the roles of autophagy. Notably, a remarkable work by Mortimore's group led to the discovery that amino acids as well as insulin and glucagon were crucial regulators of starvation-induced autophagy [6]. Subsequently, the implication of autophagy has been highlighted in various chronic liver diseases, including alcoholic liver disease, viral hepatitis, alpha1-antitrypsin deficiency, and hepatocellular carcinoma [7, 8]. Recently, several works have also pointed out the involvement of autophagy in several acute liver diseases. This review aims to summarize current knowledge on this last topic, with a particular focus on acute liver injury associated with severe anorexia nervosa.

## 2. Regulation of Starvation-Induced Autophagy

Starvation or food restriction is one of the best-known inducers of autophagy. Thus, extensive work has been carried out under this condition to study autophagy. In this stressful context, intracellular material is degraded and the resulting breakdown products are released into the cytoplasm to be used by cell metabolism [4]. In 1983, Mortimore et al. observed that mice lose about 40% of their liver protein content within 48 hrs of starvation [9]. Similarly, 4 to 5% of total protein content of isolated rat hepatocytes cultured under amino acid free conditions is degraded each hour [10]. Schworer et al. showed in rats that starvation shifts basal liver protein degradation from about 1.5%/hour (basal) to 4.5%/hour (starvation induced) [11], which correlated with autophagy kinetics determined by quantitative electron microscopy [11], leading to the concept that autophagy mediates protein degradation under nutrient deprivation [12]. Although these proteolysis rates seemed to reflect autophagic degradation, the definitive demonstration of the implication of this process was only confirmed more than 2 decades later by the use of autophagy-deficient cell models. Indeed, in isolated Atg7-deficient hepatocytes, starvation-induced proteolysis is almost completely lost [13].

Starvation-induced autophagy is regulated by several metabolic parameters including amino acid, insulin, and glucagon levels.

Experiments performed using isolated perfused liver in the absence of the potent autophagy regulators present* in vivo*, including insulin and glucagon, showed that amino acids are strong inhibitors of autophagy [12]. Indeed, half normal plasma level concentration of complete amino acid mixtures suppresses autophagy. Further investigations identified a group of 8 amino acids (leucine, tyrosine, phenylalanine, glutamine, proline, histidine, tryptophan, and methionine), including 5 essential amino acids (leucine, phenylalanine, histidine, tryptophan, and methionine), which were as effective as complete plasma mixtures for autophagy inhibition, in isolated perfused rat livers [14]. Similar results were obtained on isolated rat hepatocytes* in vitro* wherecombination of high concentrations of leucine with either histidine or glutamine inducedeffective inhibition of autophagy [15]. Leucine is by far the most efficient autophagy inhibitor and alanine, which does not have an inhibitory effect by itself, displayes a coregulatory effect [16]. Although there is evidence that most of the inhibitory effect of amino acids on autophagy occurs at the initiation step (sequestration) [6, 17], an effect on the late step (autophagosome and lysosome fusion) cannot be ruled out, since leucine at high concentration can modify lysosomal pH [18], and this might interfere with the fusion between autophagosomes and lysosomes. Furthermore, asparagine is also able to inhibit the fusion between autophagosomes and lysosomes [19]. Autophagy regulation by glutamine is indirect. A glutamine transporter, SLC1A5, is responsible for the uptake of glutamine from extracellular compartment into the cell. Glutamine is thereafter transported outside the cell by SLC7A5/SLC3A2 in exchange of essential amino acids such as L-leucine that elicit mTOR activation and subsequent autophagy inhibition [20]. The mammalian target of rapamycin (mTOR) is a central cellular metabolism protein on which several signaling pathways converge in response to changes in energy/nutritional status. mTOR stimulates protein synthesis by inducing translation of mRNA and inhibits protein catabolism by decreasing autophagy [21]. How amino acids activate mTOR is not fully understood. However, recent lines of evidence show that these molecules, when present in sufficient amounts, accumulate in lysosomes and elicit mTORC1 recruitment and activation through a lysosomal v-ATPase-Ragulator-Rag GTPase complex [22, 23]. In addition to these posttranslational effects, amino acids also modulate autophagy at the transcriptional level. During starvation, the transcription factor EB (TFEB), a master regulator of lysosomal biogenesis and autophagy, is activated, translocates into the nucleus, and drives the transcription of autophagy and lipid metabolism genes. Activity and localization of TFEB is regulated by the extracellular signal-regulated kinase 1/2 (ERK-1/2), a sensor of nutrients status [24, 25], and by mTORC1. In the presence of sufficient nutrients, TFEB interacts with a complex nutrient sensing machinery at the lysosome surface, including mTORC1 that phosphorylates TFEB at Ser211 [26, 27]. Phosphorylated TFEB is sequestered in the cytosol and is thus inactive as a transcription factor. However, during starvation, mTOR is no longer recruited at the lysosomal surface and unphosphorylated TFEB translocates to the nucleus [27].

In addition to amino acids, liver autophagy is tightly controlled by hormones. Plasma glucagon levels are increased during fasting in humans [54]. Glucagon stimulates autophagy [5]. In the presence of normal concentrations of amino acids, activation of autophagy by glucagon is maximal, whereas higher concentrations of amino acids abolish this effect [55]. Schworer et al. suggested that the stimulation of proteolysis by glucagon was a manifestation of starvation-induced autophagy. Indeed, glucagon stimulation elicits amino acids utilization for gluconeogenesis, leading to a decrease in amino acids pool. This decrease may trigger autophagy, as it mimics the effect of amino acids deprivation [55]. Although glucagon is known to activate AMPK which positively regulates autophagy [56], the mechanism of autophagy activation by glucagon remains unclear.

Insulin also plays a critical role in starvation-induced autophagy [12]. Insulin is known to activate mTOR via a class I phosphatidylinositol 3-kinase (PI3-kinase)/Akt pathway, which inhibits autophagy [57]. During fasting, plasma insulin level drops by 50% between 12 hrs and 72 hrs of fasting in humans [54, 58]. Mice also show a significant decrease in plasma insulin level after 24 hrs of starvation, while their plasma glucagon level remains relatively stable [33]. This suggests that the role of insulin level in the control of starvation-induced autophagy might be more important than that of glucagon.

## 3. Physiological Significance of Starvation-Induced Autophagy

Studies in yeast showed that autophagy is efficiently induced following 1 hour nitrogen starvation and reaches a maximal level at 3 hrs. Yeast autophagy is also induced by starvation of other nutrients such as carbon sources, sulfate, or auxotrophic amino acids [28]. Autophagy-deficient yeast cells have a loss of viability and most of them (more than 80%) die within 5 days of nitrogen starvation, indicating that starvation-induced autophagy is essential for cell viability under this stressful condition [59]. Moreover, autophagy-deficient yeasts were unable to maintain physiological levels of amino acids and to synthetize important proteins for surviving nitrogen starvation [60]. Similarly, in the eukaryote* C. Elegans,* autophagy was induced in response to nutrients shortage [32]. In the* Drosophila* larval fat body, a nutrient storage organ analogous to the vertebrate liver, starvation induced a robust autophagic response in the first 3 hrs [29].

Starvation-induced autophagy is critical during the early neonatal period in response to the sudden arrest of the transplacental supply and subsequent nutrient deprivation [61]. After birth, autophagy is immediately upregulated in various tissues, including the liver, heart, lung, diaphragm, pancreas, and the gastrocnemius muscle, and is maintained at high levels for 3–12 hrs before returning to basal levels within 1-2 days. Mice deficient for Atg5, an essential autophagy gene, die within the first day of delivery, although they appear normal at birth. Forced milk feeding of Atg5 knockout mice delayed neonates' death. This shows that autophagy is critical for survival during neonatal starvation in mammals.

Identification of key proteins regulating the autophagy machinery and the development of molecular tools to monitor autophagy* in vivo* led to a better understanding of the response of organisms to starvation. In rats as well as in mice, 24 hrs starvation increases both liver LC3II/I ratio and the number of autophagosomes assessed using electron microscopy [13, 33–35] (Table 1). Studies using GFP-LC3 transgenic mice in which the number of LC3 puncta reflects the number of autophagosomes gave similar results [30, 31] (Table 1). This model also provided evidence for differential induction patterns in several other tissues. Indeed, starvation induces autophagosome formation in the liver, skeletal muscle, heart, pancreatic acinar cells, seminal gland cells, and kidney podocytes. In most tissues, the autophagic activity reaches maximal levels within 24 hrs and then progressively decreases, whereas it further increases after 48 hrs in the heart and the soleus muscle [30]. In contrast, induction of autophagy in the brain was not observed even after 48 hrs of starvation. This might be explained by the fact that the brain is a metabolically privileged site that is supplied with glucose and ketone bodies from the liver and other tissues [62], even though brain cells are autophagy competent [63–65].

Moreover, the use of liver specific knockout models for autophagy genes unraveled a pivotal role of basal and stress-induced autophagy in the maintenance of liver cell homeostasis. Whereas starvation transiently elevates amino acid levels in the liver and the blood for 24 hrs in wild type animals, mice with liver Atg7 deficiency exhibit an impaired response to fasting, including an absence of decrease in liver protein levels and of increase in blood amino acid levels [13]. Fasting blood glucose level is also decreased in these Atg7-deficient mice [33]; this may be due to the lack of amino acids supply by autophagy for gluconeogenesis, further supporting a role of autophagy in the maintenance of blood glucose level upon starvation. In humans, although liver autophagy kinetics following starvation has not been assessed, one could speculate that autophagy is rapidly increased during fasting as in mice or rats, since plasma levels of insulin start to decrease, and those of glucagon start to increase in the first hours of fasting [54, 58].

## 4. Liver Autophagy and Anorexia Nervosa

Anorexia nervosa (AN) is characterized by a distorted perspective of body image with an intense fear of gaining weight manifesting through self-induced starvation. AN has the highest rate of mortality among eating disorders [66]. Two main subtypes of AN are recognized: restricting type and binge-eating/purging type. Average prevalence of AN is of 0.3% in young women [67] and might be up to 4% with a broader definition, close to DSM-5 criteria [68]. AN can be associated with several medical complications, including cardiovascular complications (bradycardia and hypotension), gastrointestinal problems (lack of food intake induces reflex hypofunctioning of the colon and subsequent constipation), endocrine and electrolytes abnormalities, amenorrhea in women [69, 70], and liver blood tests abnormalities [36–53] (Table 2). Mild increase in serum transaminases levels (<200 IU/L) is observed in up to 75% of AN patients [52]. Marked increases (>200 IU/L) are less common (Table 2) [46, 71–78]. Interestingly, several independent groups observed that serum transaminases levels inversely correlate with body mass index (BMI) [46, 51, 53], suggesting a role of nutritional status in the liver changes of these patients. However, understanding of the mechanisms of these abnormalities is hampered by the absence of available description of liver histological or ultrastructural changes.

Although much less common, severe liver insufficiency associated with AN has been better investigated [49]. A series of 12 patients with acute liver insufficiency (prothrombin index <50% and/or an international normalized ratio >1.7) and AN as the only cause for acute liver injury has been analyzed. All patients had severe AN attested by a BMI systematically equal to or less than 13 kg/m^2^ and by severe hypoglycemia and coma at admission in half of them. Serum transaminases levels were highly increased in all patients (average 2000 IU/L) suggesting severe liver injury. Liver biopsies were available in all patients. Surprisingly, liver histological analysis as well as TUNEL staining disclosed no or rare features of necrosis or apoptosis. On electron microscopy, hepatocytes showed numerous autophagosomes, as well as a low density of organelles and of glycogen. Moreover, some hepatocytes presented morphological characteristics of autophagic cell death (also called type II cell death). This aspect was not observed in patients with other causes of acute liver insufficiency. These results support the view that hepatocytes autophagic death was the leading pathway of acute liver injury in patients with severe AN. This may explain the increase in aminotransferases levels in the absence of hepatocytes necrosis on histology, since autophagic cell death is associated with cytoplasmic membrane permeability, allowing the release of transaminases in the blood [49]. Patients management with controlled enteral supplementation, plasma glucose, and electrolytes correction led to rapid improvement in liver function. None of them developed hepatic encephalopathy, and all patients with initial cardiac dysfunction recovered within one month. This beneficial effect of refeeding further supports the role of severe starvation and subsequent autophagic cell death in acute liver injury in these patients.

**Figure 1:**
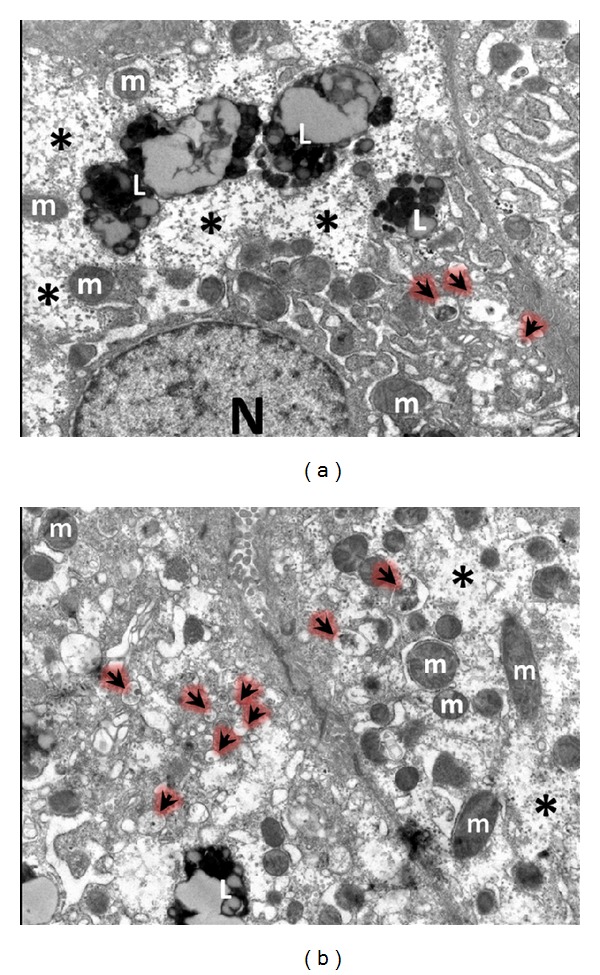
Electron microscopy pictures of hepatocytes from patients with severe anorexia nervosa. Hepatocytes show low density of organelles in the cytoplasm, glycogen depletion (∗), and autophagosomes sequestering cytoplasmic material (arrows), N: nucleus; m: mitochondria; L: mature lysosomes.

Altogether, we can speculate that starvation-induced autophagy in AN plays a dual role. During the first phase of weight loss, liver blood tests abnormalities are moderate suggesting that autophagy can cope with nutrient deprivation. During that period, autophagy is protective and prevents cell death. When starvation continues and BMI reaches a critical level equal or less than 13 kg/m², excessive activation of autophagy leads to hepatocyte cell death and liver insufficiency (Figure 1).

## 5. Autophagy in Acute Liver Injury

**Figure 2:**
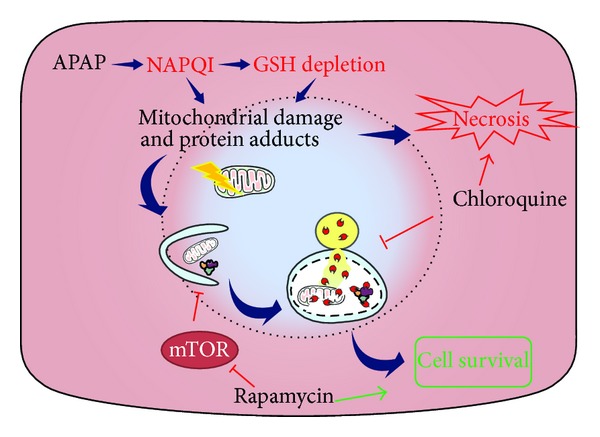
Protective role of autophagy in APAP-induced hepatotoxicity. APAP is metabolized in hepatocytes to generate NAPQI, which depletes GSH stores and induces mitochondrial damage by generating protein adducts, leading to hepatic necrosis. Autophagy is induced as a defense mechanism and promotes cell survival by removing damaged mitochondria and decreasing oxidative stress. Pharmacological activation of autophagy promotes cell survival while its inhibition favors cell death, APAP: acetaminophen; NAPQI: N-acetyl-p-benzoquinone imine; GSH: glutathione; mTOR: mammalian target of rapamycin.

Recent studies highlighted the involvement of autophagy in drug-induced hepatotoxicity. Overdose of acetaminophen (APAP), a widely used antipyretic and analgesic drug, is the first cause of acute liver failure in humans [79]. The mechanisms leading to APAP-induced liver injury are well documented. In the liver, therapeutic doses of APAP are mainly excreted into the bile or the blood after glucuronic acid and sulfate conjugation. A small amount of the drug is metabolized to N-acetyl-p-benzoquinone imine (NAPQI) by cytochrome P450 enzymes, mainly via CYP2E1 isoform. NAPQI, which is highly electrophilic, reacts with glutathione (GSH) to form a GSH adduct. In case of APAP overdose, GSH stores are exhausted and NAPQI binds to cellular, including mitochondrial, proteins leading to mitochondrial damages and necrotic cell death [80]. As a defense mechanism against necrosis, APAP induces autophagy to remove damaged mitochondria [81]. Interestingly, mitochondria are frequently seen within APAP-induced autophagosomes, and expression level of mitochondrial proteins is decreased, supporting the role of mitophagy in the removal of damaged mitochondria. Moreover, autophagy inhibition by chloroquine or 3-methyladenine exacerbates APAP-induced necrosis, whereas induction of autophagy with rapamycin completely blocks it, further supporting a protective role of autophagy in APAP-induced liver injury [81] (Figure 2). Consistent with these data, studies performed by Igusa et al. using inducible liver Atg7-deficient mice indicated that loss of autophagy promoted APAP-induced reactive oxygen species, mitochondrial damage, and subsequent liver injury [82]. However, mice with a constitutive hepatocyte specific deletion in Atg5 displayed resistance to APAP overdose [83]. In these constitutive Atg5 deficient mice, compensatory increase in hepatocytes proliferation and in basal GSH levels as well as faster recovery of GSH content after APAP insult mediated by persistent activation of Nrf2 could account for this apparent discrepancy. Indeed, prolonged loss of autophagy increases levels of p62 leading to stabilization of Nrf2 and in turn to transcriptional activation of Nrf2 target genes, including antioxidant proteins and detoxifying enzymes [84]. These discrepancies between inducible and constitutive genetic deletions indicate that caution should be taken when working with genetic models of autophagy deficiency, as discussed elsewhere [85, 86]. There is to date no data on autophagy level in the liver of patients with APAP overdose. Electron microscopy analysis of liver samples from patients could be useful to confirm what has been observed in mice [81]. Chronic exposure to alcohol decreases autophagic flux by inhibiting the fusion of autophagosomes with lysosomes [87]. This may explain why chronic consumption to alcohol favors APAP hepatotoxicity [88, 89]. Besides induction of autophagy, APAP also induces the formation of mitochondrial spheroids* in vivo* [90], which are ring-like spherical structures with lumen surrounded by mitochondrial membranes that can contain cytoplasmic material. Formation of mitochondrial spheroids in response to oxidative stress is inversely correlated with Parkin expression and requires mitofusins [90]. However, the exact mechanisms by which APAP induces mitochondrial spheroids remain to be elucidated. Ni et al. suggested that posttranslational modifications of Parkin due to increased nitric oxide (NO) and reactive nitrogen species by APAP may promote mitofusin-mediated formation of mitochondrial spheroids [91]. Although the physiological significance of mitochondrial spheroids formation in response to APAP is not clear, this mechanism may represent an alternative defense route against APAP-induced liver injury. Further work is needed to address this issue.

**Figure 3:**
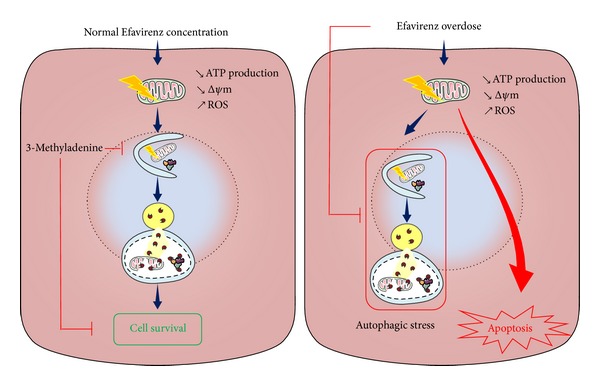
Role of autophagy in Efavirenz-induced hepatotoxicity. Clinically relevant concentration of Efavirenz induces mitochondrial dysfunction, which in turn induces autophagy, thereby promoting cell survival. However, higher concentration of Efavirenz is associated with inhibition of autophagic flux, which seriously compromises cell survival, ATP: adenosine triphosphate; Δ*ψ*m: mitochondrial membrane potential; ROS: reactive oxygen species.

Efavirenz, a nonnucleoside reverse transcriptase inhibitor widely used to treat HIV infections can be hepatotoxic in some patients [92]. The molecular pathogenesis of this effect involves mitochondrial dysfunction and subsequent decrease in ATP production and mitochondrial membrane potential and increase in reactive oxygen species generation [93]. At clinically relevant concentrations, Efavirenz induces mitochondrial damage and triggers mitophagy as a rescue mechanism. The beneficial effect of mitophagy is supported by the fact that pharmacological inhibition of autophagy enhances Efavirenz-induced cell death [94]. At higher concentrations, corresponding to those observed in slow metabolizing patients [95], Efavirenz blocks autophagic flux, leading to an increase in mitochondrial damage and eventually to cell death [94] (Figure 3). This complex concentration-dependent dual effect of Efavirenz on hepatocytes autophagy may be involved in other hepatotoxic drugs mechanisms that interfere with mitochondrial function.

The role of autophagy has been investigated in two other models of acute liver injury, namely, the concanavalin A (Con-A) and the lipopolysaccharide/D-galactosamine models. Con-A induces hepatitis by T cell-dependent and T cell-independent mechanisms. The former mechanism induces hepatocyte apoptosis whereas the latter leads to hepatocyte autophagic cell death [96]. Indeed, intravenous injection of Con-A in SCID/NOD mice, that is, mice with a defect in lymphocytes function, induced an acute hepatitis associated with an increased autophagy as demonstrated by the increased LC3I conversion to LC3II [96]. Con-A also induces cell death in hepatoma cell line by a mechanism involving mitochondrial membrane permeability, BNIP3 induction, and LC3-II generation. Concanavalin A-induced cell death could be partially inhibited by either 3-methyladenine or knockdown of BNIP3 and LC3 by siRNA, suggesting that autophagy is involved in its effect [97]. Not only hepatocytes, but also liver endothelial cells can undergo autophagic cell death following Con-A exposure* in vitro* and in mice [98]. Altogether, these data highlight a deleterious effect of Con-A-induced autophagy on hepatic cells. By contrast, induction of liver autophagy in the lipopolysaccharide/D-galactosamine model seems to be hepatoprotective. Indeed, autophagy was rapidly induced in both wild type and pregnane X receptor (PXR) knockout mice after lipopolysaccharide/D-galactosamine insult. However, this increase was only transient in the latter group, and autophagy level rapidly dropped. This significant reduction of autophagy in PXR knockout mice was associated with a greater liver injury, characterized by increased alanine aminotransferase, hepatocyte apoptosis, necrosis, and hemorrhagic liver injury [99].

## 6. Conclusion

Increasing evidence demonstrates that autophagy plays a critical role in acute liver injury related to severe anorexia nervosa and to drug overdose. Increased liver autophagy level is a common feature of these diseases. Autophagy is mainly hepatoprotective. In anorexia nervosa, autophagic cell death occurs only when body mass index reaches a critically low level. After APAP or Efavirenz exposure, autophagy removes damaged mitochondria, and liver injury appears only when this process is either blocked by other factors or overwhelmed. Whether molecules stimulating autophagic flux are beneficial in acute liver injury remains to be determined.

## Figures and Tables

**Table 1:** Starvation-induced autophagy in different experimental models.

Author, year	Model	Techniques used	Time point
Takeshige et al., 1992 [28]	*S. cerevisiae *	Electron microscopy: increase in the number of autophagosomes and delivery to the vacuole	1 hrs–3 hrs
Scott et al., 2004 [29]	Drosophila fat body	Electron microscopy: increase in the number of autophagosomes	3 hrs
Mizushima et al., 2004 [30]	GFP-LC3 transgenic mice	Fluorescence microscopy: increase in the number of LC3-GFP dots (24 h) and then return to basal level (48 h)	24 hrs–48 hrs
Komatsu et al., 2005 [13]	Atg7F/+:Mx1-Cre mice	Electron microscopy: increase in the number of autophagosomes Western blot: increase in LC3II/I ratio	24 hrs
Martinet et al., 2006 [31]	GFP-LC3 transgenic mice	Fluorescence microscopy: increase in the number of LC3-GFP dots	24 hrs–48 hrs
Hansen et al., 2008 [32]	LGG-1-GFP transgenic *C. Elegans* Eat-2 mutants	Fluorescence microscopy: increase in the number of LGG-1-GFP foci	60 hrs
Ezaki et al., 2011 [33]	C57BL/6J mice	Electron microscopy: increase in the number of autophagosomes Western blot: increase in LC3II/I ratio	24 hrs 3 hrs–18 hrs
Uddin et al., 2011 [34]	C57BL/6 mice	Western blot: increase in LC3II/I ratio	12 hrs–24 hrs–36 hrs–48 hrs
Skop et al., 2012 [35]	Wistar rats	Western blot: increase in LC3II/I ratio	24 hrs

GFP: green fluorescent protein; LC3: microtubule-associated protein 1 light chain 3 alpha; LGG-1: LC3, GABARAP, and GATE-16 family; Mx-1: myxovirus (influenza virus) resistance 1.

**Table 2:** Studies assessing liver blood tests in patients with anorexia nervosa.

Author, year	Body mass index (kg/m^2^)	Percentage of patients with increased serum transaminases levels
Cravario et al., 1974 [36]	14.4	4% (*N* = 27)
Kanis et al., 1974 [37]	15	0% (*N* = 24)
Milner et al., 1985 [38]	—	45% (*N* = 42)
Mira et al., 1987 [39]	15.9	9% (*N* = 22)
Palla and Litt, 1988 [40]	—	33% (*N* = 24)
Umeki, 1988 [41]	—	59% (*N* = 27)
Hall et al., 1989 [42]	—	32% (*N* = 31)
Waldholtz and Andersen, 1990 [43]	—	0% (*N* = 13)
Sherman et al., 1994 [44]	—	26% (*N* = 19)
Mickley et al., 1996 [45]	—	7% (*N* = 282)
Ozawa et al., 1998 [46]	13.2	29% (*N* = 101)
Miller et al., 2005 [47]	16.8	12% (*N* = 214)
Montagnese et al., 2007 [48]	15.6	14% (*N* = 97)
Rautou et al., 2008 [49]	11.3	66.6% (*N* = 12)
Fong et al., 2008 [50]	18	26% (*N* = 53)
Tsukamoto et al., 2008 [51]	15.2	52% (*N* = 25)
Gaudiani et al., 2012 [52]	13.1	76% (*N* = 25)
Hanachi et al., 2013 [53]	12	56% (*N* = 126)

Cumulated (mean)	14.6	24% (278/1158)

## Abbreviations and Acronyms

Akt: Protein kinase B
AN: Anorexia nervosa
APAP: Acetaminophen
Atg: Autophagy-related gene
BMI: Body mass index
BNIP3: BCL2/adenovirus E1B 19 kDa protein-interacting protein 3
Con-A: Concanavalin A
CYP2E1: Cytochrome P450 2E1
DSM: Diagnostic and statistical manual of mental disorders
ERK 1/2: Extracellular signal-regulated kinases 1/2
GFP: Green fluorescent protein
GSH: Glutathione
LC3: Microtubule-associated protein 1 light chain 3 alpha
LGG-1: LC3, GABARAP, and GATE-16 family
mTOR: Mammalian target of rapamycin
mTORC1: Mammalian target of rapamycin complex 1
Mx-1: Myxovirus (influenza virus) resistance 1
NAPQI: N-acetyl-p-benzoquinone imine
Nrf-2: Nuclear factor erythroid 2-related factor 2
PI3K: Phosphatidylinositol-4,5-bisphosphate 3-kinase
PXR: Pregnane X receptor
SCL1A5: Solute carrier family 1 (neutral amino acid transporter), member 5
SCL3A2: Solute carrier family 3 (amino acid transporter heavy chain), member 2
SCL7A5: Solute carrier family 7 (amino acid transporter light chain, L system), member 5
TFEB: Transcription factor EB
v-ATPase: Vacuolar—type H+—ATPase.

## Data Sources and Searches

The authors searched PUBMED (1960–2014) for studies on autophagy and liver physiology and pathology by using combinations of the terms: autophagy, autophagosome, liver, starvation, acute liver injury, anorexia nervosa, acetaminophen, and Efavirenz. Relevant papers were acquired as abstracts or full text. The authors also reviewed publications in personal reference lists and citation sections of the recovered articles.
